# Low-dose CT-based implant motion analysis is a precise tool for early migration measurements of hip cups: a clinical study of 24 patients

**DOI:** 10.1080/17453674.2020.1725345

**Published:** 2020-02-14

**Authors:** Cyrus Brodén, Olof Sandberg, Olof Sköldenberg, Hampus Stigbrand, Mari Hänni, Joshua W Giles, Roger Emery, Stergios Lazarinis, Andreas Nyström, Henrik Olivecrona

**Affiliations:** a Department of Surgery and Cancer, Imperial College London, London, UK;; b Department of Clinical Sciences, Danderyd Hospital, Karolinska Institutet, Stockholm, Sweden;; c Sectra, Linköping, Sweden;; d Department of Orthopedic Surgery, Länssjukhuset, Gävle, Sweden; Center for Research and Development, Uppsala University/County Council of Gävleborg, Sweden;; e Department of Surgical Sciences, Section of Radiology, Uppsala University Hospital, Uppsala, Sweden;; f Department of Mechanical Engineering, University of Victoria, Victoria, BC, Canada;; g Department of Orthopaedic Surgery, St Mary’s Hospital, London, UK;; h Department of Orthopedics, Institute of Surgical Sciences, Uppsala University Hospital, Uppsala, Sweden;; i Department of Molecular Medicine and Surgery, Karolinska Institutet, Stockholm, Sweden

## Abstract

Background and purpose — Early implant migration is known to be a predictive factor of clinical loosening in total hip arthroplasty (THA). Radiostereometric analysis (RSA) is the gold standard used to measure early migration in patients. However, RSA requires costly, specialized imaging equipment and the image process is complex. We determined the precision of an alternative, commercially available, CT method in 3 ongoing clinical THA studies, comprising 3 different cups.

Materials and methods — 24 CT double examinations of 24 hip cups were selected consecutively from 3 ongoing prospective studies: 2 primary THA (1 cemented and 1 uncemented) and 1 THA (cemented) revision study. Precision of the CT-based implant motion analysis (CTMA) system was calculated separately for each study, using both the surface anatomy of the pelvis and metal beads placed in the pelvis.

Results — For the CTMA analysis using the surface anatomy of the pelvis, the precision ranged between 0.07 and 0.31 mm in translation and 0.20° and 0.39° for rotation, respectively. For the CTMA analysis using beads the precision ranged between 0.08 and 0.20 mm in translation and between 0.20° and 0.43° for rotations. The radiation dose ranged between 0.2 and 2.3 mSv.

Interpretation — CTMA achieved a clinically relevant and consistent precision between the 3 different hip cups studied. The use of different hip cup types, different CT scanners, or registration method (beads or surface anatomy) had no discernible effect on precision. Therefore, CTMA without the use of bone markers could potentially be an alternative to RSA to measure early migration.

Early migration within the first 2 years after implantation of hip and knee implants is a strong indicator of future clinical loosening (Kärrholm 2012, Pijls et al. 2012, Klerken et al. 2015). The gold standard to assess early migration in orthopedic implants is radiostereometry (RSA) (Valstar et al. 2005). There are some limitations of the standard RSA technique such as the dependency on using tantalum beads, the specialized laboratories and expensive imaging equipment, the need for specially trained personnel to conduct the examinations, and the unavoidable loss of data due to tantalum beads being occluded or hidden (Kaptein et al. 2005). Over the last decades, improvements in CT hardware and software have increased CT scan quality while also reducing the effective dose. This combined with increases in computer processing power means that CT now can form the basis for an alternative method to measure implant migration while mitigating the aforementioned limitations of RSA. Early phantom studies and clinical pilots have indicated that with methods using CT scans, a precision comparable to that of RSA can be achieved (Brodén et al. 2016, Olivecrona et al. 2016, Scheerlinck et al. 2016)

Lately, commercial image-registration software named “CT-based implant Motion Analysis” (CTMA, Sectra, Linköping) has been developed to assess implant migration using standard clinical CT scanners. This article explores the CTMA tool in a clinical setting, using 3 different types of hip cups (2 cemented and 1 uncemented) drawn from different centers in Sweden to evaluate the precision of this technique.

We estimated the precision of the CTMA technique in 2 different settings: first, omitting bone markers relying solely on the pelvic anatomy for the image registration; and second, using bone markers for the image registration.

## Materials and methods

### Subjects and clinical setup

Study subjects were included from 3 ongoing clinical studies in different regions within Sweden: Uppsala University Hospital in Uppsala, Danderyds Hospital in Stockholm, and Gävle Hospital in Gävle. 24 hip implants were included in this study. For every hip, 2 consecutive CT examinations were conducted (i.e., a double CT examination). A different CT scanner, CT protocol, and implant was used at each hospital as specified below.

### 1. Uppsala University Hospital

#### Study setting

**Figure 1.**
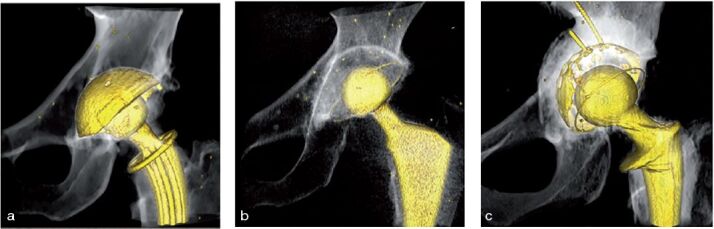
Implant types used in the studies: (a) the uncemented TOP cup used in the Uppsala study, (b) a cemented Muller Exceed ABT polyethylene cup from the Danderyd study, (c) the graft-compressing titanium shell and a cemented Lubinus cross-linked UHMWP polyethylene cup used in the Gävle study.

CT examinations from 5 randomly selected patients were included from an ongoing prospective cohort study. The study explores bone mineral density around a hip stem and migration of hip components investigated by RSA. Patients received an uncemented hip prosthesis with the CFP (Collum Femoris Preserving) stem and the TOP (Trabeculae-Oriented Pattern cup), provided by Waldemar Link Gmbh (Hamburg, Germany) (Figure 1) Perioperatively, beads were inserted in the pelvic and femoral bones.

RSA was used to analyze component migration, and clinical follow-up was performed at 3, 12, and 24 months postoperatively. At the 7-year follow up, a CT double examination was performed. More details of this study can be found in the 2-year follow-up publication (Lazarinis et al. 2013).

#### CT examinations

The 2 consecutive CT scan were obtained with a 2x64, dual source CT scanner (Somatom Definition Flash, Siemens, Forchheim, Germany). Between scans the patient was repositioned on the CT table. Details of CT protocol settings are described in Table 1.

**Table 1.** CT settings from 3 clinical trials. A bone filter has been used for all scans without any metal artefact reduction algorithm.

Site of CT scanner	kVp ^a^ (V)	Tube current (mA)	Exposure (mAs)	Slice thickness (mm)	Increments (mm)	Pitch	Rotation time (s)
Uppsala University Hospital **^b^**	120	23	23	0.6	0.6	0.9	1
Danderyd’s Hospital **^c^**	120	10	10	0.625	0.312	0.98	1
Gävle Hospital **^d^**	120	80–500 ^e^	55	0.5	0.5	0.81	0.5

**^a^**kVp: Kilovoltage peak is the peak voltage applied to the X-ray tube.

**^b^**Somatom Definition Flash, Siemens, Forchheim, Germany.

**^c^**Discovery CT750HD, GE Healthcare, Chicago, IL, USA.

**^d^**Aquilon One CT scanner (Toshiba).

**^e^**Dynamic tube current.

### 2. Danderyds Hospital

#### Study setting

CT examinations from 9 randomly selected patients were included from an ongoing randomized study. The study compared proximal migration of 2 types of cemented cups: an Argon gas-sterilized Polyethylene (PE) group and a Vitamin E treated PE group (Muller Exceed ABT, Biomet, Warsaw, IN, USA) (Figure 1). Patients who were included had a diagnosis of primary osteoarthritis planned for total hip arthroplasty. Patients were followed with RSA at 3 months and at 1, 2, and 5 year(s) postoperatively. A double CT examination was also performed at 3 months postoperatively. Tantalum beads were inserted in the pelvis and cup. For more details, the study protocol has been published (Sköldenberg et al. 2016).

#### CT examinations

2 consecutive CT scans were obtained with a 128-detector CT scanner (Discovery CT750HD, GE Healthcare, Chicago, IL, USA). Between scans the patient was repositioned on the CT table. The CT settings are described in Table 1.

### 3. Gävle Hospital

#### Study setting

CT examinations from 10 randomly patients were included from an ongoing revision THA study on early migration. Included patients had THA revision surgery due to cup loosening and acetabular osteolysis. Exclusion criteria were systemic diseases affecting the skeleton, and/or medication with known effect on bone metabolism. The mean age at surgery was 73 years (49–87). Acetabular reconstruction was performed using an impaction bone graft with a titanium compressing shell and a cemented cup (Lubinus cross-linked UHMW-PE, Waldemar Link, Hamburg, Germany) (Figure 1). Tantalum beads were spread in the pelvis. CT scans were performed postoperatively, after 6 weeks, and after 2 years.

#### CT examinations

2 consecutive CT scans were obtained at 6 weeks postoperatively with a 160 detector Aquilon One CT scanner (Toshiba). In between the double CT examinations the patient was repositioned. Details of CT protocol settings are described in Table 1.

### The definition of precision

The precision of a measurement is defined by the “degree to which repeated measurement under unchanged conditions show the same results” (Sköldenberg et al. 2014). In the datasets used in this study, a double CT examination was performed in which 2 CT scans were conducted on the same day for each patient. The CT scans were performed under the same conditions consecutively with the patient standing up in between. The assumption is that no movement between cup and pelvis occurs between these 2 examinations. Therefore, any measurement other than zero is attributed to the errors of the method.

### Image analysis and evaluation procedure

To assess the precision of the CTMA system, an estimation of the random errors of the method was made.

**Figure 2.**
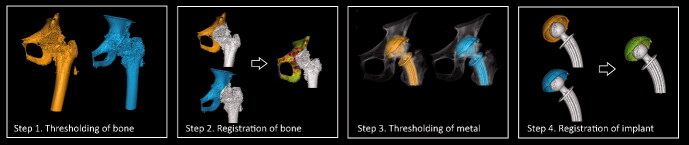
Processing schematic for CTMA. (Step 1) First an optimization of bone thresholding is performed manually. (Step 2) Thereafter the pelvic bone is defined here by the surface of the pelvic anatomy; a first registration is subsequently performed. (Step 3) A manual thresholding of metal is performed. (Step 4) The implant rigid body (the cup) is defined. The user indicates the region of interest for the second registration in the 2 datasets, i.e., the implant. Next the second registration occurs. Green color indicates a successful registration.

Since no movement had occurred between cup and pelvis in between the 2 CT examinations in 1 double examination, the measured migration by the CTMA system reflected the random errors and therefore the precision of the CTMA method. To assess the measured motion of the cup relative to the pelvis by the software the following steps were performed (Figure 2):

1. The CT volumes were imported into the CTMA system, and an optimization of bone or bead thresholding was performed manually. The same threshold was used for all examinations from a given cohort.
2. The reference rigid body (pelvic bone) was defined in 2 separate CT examinations and registered to obtain a visual overlap of the bone of the 2 volumes.
3. A manual thresholding of metal was performed using the same setting for all patients from a given cohort.
4. The implant rigid body (the cup) was defined in 2 separate CT examinations and registered to obtain a visual overlap of the implant of the 2 volumes.

The software calculated the motion that has occurred for the implant relative to the pelvic bone between these 2 CT volumes described in the CT-based coordinate system. It resulted in a visual output in the form of registered 3D volumes as well as numerical migration values expressed in 6 degrees of freedom (rotations around and translation along x, y, z, in a CT DICOM coordinate system).

The CTMA procedure described earlier (steps 1–4) was repeated twice. The first series was performed by defining the reference rigid body using the surface anatomy of the pelvis without the use of bone markers (Figure 2). The second series was performed using tantalum beads in the pelvic bone to define the reference rigid body.

For both series, the implant rigid body was defined either by the metallic surface of the implant or the metallic threads and/or beads in the implant depending on the implant type.

During the analysis, the consistency of the registered rigid bodies was verified manually with a color-coded feedback mechanism that illustrates any change in the transformation of either rigid body due to deviation of tantalum beads, bone morphology changes, or implant deformation. Green color indicated a successful registration.

### Radiation

The effective dose of the CT method was estimated for each dataset. The dose length product (DLP) indicates the overall energy delivered along the scan length. DLP is then multiplied by the constant (k) of the pelvis that provides the conversion from mGy · cm to mSv, which is the unit of effective dose based on the tissue exposed (AAPM, 2008).

### Statistics

Statistical analyses were performed in SPSS Statistics, version 25 (SPSS Hong Kong 1804, Westlands Centre, Westlands Road, Quarry Bay, HK). Data from each type of implant were tested separately for normality using the Kolmogorov–Smirnoff test.

We estimated the precision of the method using the standard deviation of double measurements and the critical values that encompass 95% of Student’s t-distribution with n-1 degrees of freedom, where n represents the number of patients (Sköldenberg and Odquist 2011).

### Ethics, funding, and potential conflict of interest

The Uppsala study was approved by the ethics committee of Uppsala University (Dnr 2007/105/2). The Karolinska study was approved by the ethics committee of Karolinska Institute (No. 2011/2003-31/1). The Gävle study was approved by the ethics committee of Uppsala University (Dnr 2015/228).

No funding was received for this study. HO and CB have received consultancy fees from Sectra; Olof Sandberg is a full-time employee at Sectra. 

## Results

For the CTMA technique using the surface of the pelvic anatomy, the precision ranged between 0.07 and 0.31 mm in translation and between 0.20° and 0.39° for rotation (Table 2). For the CTMA analysis using beads in the bone, the precision ranged between 0.08 and 0.20 mm in translation and between 0.20° and 0.43° for rotation (Table 3).

**Table 3.** Precision of CTMA of different cups using bone markers for registration

Hospital	Translation, mm					Rotation, °		
	n	x	y	z	TT	x	y	z
Uppsala University Hospital	5	0.11	0.09	0.08	0.03	0.43	0.23	0.22
Danderyd’s Hospital	9	0.08	0.13	0.13	0.13	0.25	0.21	0.29
Gävle Hospital	10	0.10	0.20	0.14	0.12	0.20	0.22	0.26

TT: total translation.

**Table 2.** Precision of CTMA of different cups using the pelvic anatomy without beads for registration

Hospital	Translation, mm					Rotation, °		
	n	x	y	z	TT	x	y	z
Uppsala University Hospital	5	0.07	0.13	0.31	0.20	0.37	0.22	0.39
Danderyd’s Hospital	9	0.23	0.11	0.08	0.16	0.31	0.28	0.29
Gävle Hospital	10	0.12	0.31	0.15	0.22	0.28	0.20	0.23

TT: total translation.

For the visual feedback, no movement within the prosthesis and the bone was visualized between any of the sets of 2 CT scans forming a double examination, which agrees with our assumption that no true migration had occurred during patient repositioning in the CT scanner. The effective dose of CT scans differed between the clinical trials: the mean effective dose was 0.7 mSv for Uppsala; 0.2 mSv for Danderyd’s Hospital and 2.3 mSv for Gävle Hospital. Artefacts could be detected in the CT scans, but no movement artefacts were present. 

## Discussion

This study determined the clinical precision of the CTMA method in 3 ongoing clinical THA studies with variable protocols (e.g., implant type, CT scanner, and CT protocol). The precision of the technique using pelvic anatomy ranged between 0.07 and 0.31 mm in translation and 0.20° and 0.39° for rotation while the precision of the technique using beads implanted in bone ranged between 0.08 and 0.20 mm in translation and between 0.20° and 0.43° for rotations. Precision values of standard RSA have been estimated to range between 0.15 and 0.60 mm for translation and between 0.3° and 2° for rotations in the lower limb, which is slightly less precise than our findings in this study (Kärrholm et al. 1997).

Micromotion of more than 1.2 mm has been linked to higher revision risk (Kärrholm et al. 1994). Our results indicate that CTMA is more precise than this threshold, making it capable of detecting clinically important micromotion. The precision of CTMA of the 3 different cups used in this study did not differ markedly, despite there being differences in geometry and implant material. In addition, each type of implant was scanned with a different CT scanner and CT scanning protocol. The technique also demonstrated an insensitivity to x, y, and z directions as the precision values differed only slightly between x, y, and z for translation and rotation.

Scheerlink et al. (2016) previously developed a CT method to assess early migration in hip prostheses in both a clinical and an experimental setting. Their results in vitro were as accurate and precise as RSA in the lower limb, with a precision better than 0.09 mm and 0.14°. In a clinical setting, Scheerlink et al. presented values such as mean absolute error to estimate precision. Since our methodology for estimating precision was different in this CTMA study, the comparison is difficult. However, while our study reports results using low-dose CT (which is more challenging but crucial for widespread clinical application), the Scheerlink paper reports exposure of the CT scans of 3.1 to 8.2 mSv, which limits the use of their technique. This is an important difference as the European Commission, in its guidance on exposure in medical and biomedical research, “Radiation protection 99” (European Commission, 1998), gives approximate values of between 0.5 and 10 mSv as the target per person for an entire study of this type. In contrast, our method achieved substantially lower radiation doses across different CT scanners and protocols (0.2–2.3 mSv). The quality of the CT images improved with the higher dosage and the segmentation of the image was easier; however, all collected data could easily be used for the CTMA analysis and the precision did not improve with higher dosage. This effective dose could be compared to RSA examination that delivers a dose of 0.15 mSv and a normal pelvic radiograph of 0.7 mSv (Valstar 2001, Boettner et al. 2016) However, in practice, RSA examination involves additional retakes and the effective dose would often be higher than the quoted 0.15 mSv.

This study only included acetabular components, thus further studies are needed to evaluate whether the reported precision level is consistent for other implants. For example, the femoral stem has a larger volume, is more asymmetric, and the material is metallic. These three factors should diminish registration errors. At the same time the femur is a more elongated and symmetrical bone compared with the pelvis. As a result of these conflicting factors, it is probably not possible to extrapolate from the current results.

Another aspect to consider in future studies is the efficacy of the CTMA software in following component migration over time. The registration process of the bone could, in theory, be more complicated because of the changes in the bone morphology over time. However, the software has a feature that identifies differences between the CT volumes and, for the purposes of registration, uses only portions of the bone morphology that are common in each scan.

An implant motion analysis system based on low-dose CT scans and free of bone tantalum markers opens up the possibility for simpler implant migration studies. This could be used to create more rigorous quality control of new implants before widespread market introduction, while minimizing impediments to innovation.

The CTMA addresses some of the limitation of RSA such as the need for special personnel to perform the image acquisition, the need for an expensive RSA laboratory, and the problems of obscured markers. However, CTMA expertise is needed to perform some of the manual steps of the image analysis. Each image registration takes between 10 and 20 minutes to perform.

In conclusion, our study demonstrates in 3 ongoing clinical studies that CT-based migration measurement achieves a clinically relevant precision without the need for bone markers. It demonstrates that this technique can be used on different types of cup implants using CT volumes with different CT scanners at different effective doses and still maintain the clinically relevant precision needed to follow early migration of cups in hip arthroplasty.
